# The measurement of stiffness of uterine smooth muscle tumor by elastography

**DOI:** 10.1186/2193-1801-3-294

**Published:** 2014-06-11

**Authors:** Shigenori Furukawa, Shu Soeda, Takafumi Watanabe, Hiroshi Nishiyama, Keiya Fujimori

**Affiliations:** Department of Obstetrics and Gynecology, Fukushima Medical University School of Medicine, 1-Hikarigaoka, Fukushima City, 960-1295 Japan

**Keywords:** Uterine Leiomyosarcoma, Uterine Leiomyoma, Elastography, Virtual Tissue Imaging (VTI), Virtual Tissue Quantification (VTQ)

## Abstract

Leiomyoma shows various diagnostic images, often making it difficult to differentiate from leiomyosarcoma. Recently, the utility of elastography has been reported for the differentiation of superficial tumors. We attempted to diagnose two cases of uterine smooth muscle tumors by elastography.

One case was strongly suspected of leiomyosarcoma, and the other case had been diagnosed with leiomyoma. We preoperatively performed virtual tissue imaging (VTI) and virtual tissue quantification (VTQ). In VTQ, we measured shear wave velocity (Vs) five times at each point that ROI was placed. In case of suspected leiomyosarcoma, we attached the tip of convex probe 2 cm below the navel, perpendicular to the floor and measured Vs. In case of leiomyoma, we placed four ROIs randomly in leiomyomas for VTQ. For the case of suspected leiomyosarcoma, Vs and pathological findings from the VTQ were comparably examined. Significant differences were observed in the Vs in the leiomyosarcoma case, whereas not in the leiomyoma case. The comparison of VTQ and pathological findings for the case of leiomyosarcoma indicated high viability in the region where the highest Vs was measured, and strong necrosis in the region with the lowest Vs. These findings suggest that VTQ is useful for diagnosing uterine smooth muscle tumors.

## Case report

### Case 1

A seventy-year-old woman visited our hospital complaining of worsening abdominal oppression which started several months ago. She had been found to have a leiomyoma at a medical checkup and followed up because of no tendency of increase. A pelvic mass of 20 cm in major axis, which reached the height of the navel, was palpated on examination. No abnormality in neither vaginal nor endometrial cytology was detected. Tumor markers CEA, CA19-9 and CA125 were within normal ranges on a blood test and a serum biochemical test revealed that the LDH level was slightly elevated (320 IU/l), but no other abnormalities were detected. MRI revealed a huge heterogeneous uterine tumor, strongly suggesting a sarcoma. CT showed no distant metastasis or peritoneal dissemination. Hysterectomy and bilateral adnexectomy was performed. The tumor was huge and contained necrotic components and solid components as seen in the preoperative imaging. Histopathological findings revealed smooth muscle cells with more than 10/10HPF mitosis and coagulation necrosis, leading to a diagnosis of leiomyosarcoma.

**Figure 1:**
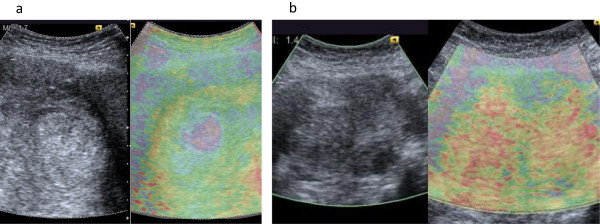
**Ultrasound images of uterine leiomyosarcoma (a) and leiomyoma (b).** Left: US B-mode image Right: VTI. The results of gray-scale and VTI are shown in Figure 1. Irregular distribution of blue, yellow, green and red was seen in VTI suggesting a heterogeneous inner structure. Notable blue was present in high echoic spots shown on gray-scale imaging. In VTQ study of the leiomyosarcoma, four ROIs were placed.

**Figure 2:**
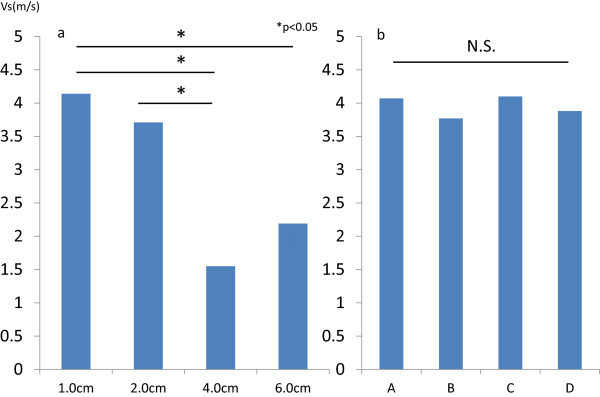
**Vs measured in the uterine leiomyosarcoma (a) and leiomyoma (b).** Significant differences were observed among the four points measured in the leiomyosarcoma, whereas no significant difference was observed among the four points measured in the leiomyoma. In bilateral graph, X-axis shows the points that VTQ was examined. In **(a)**, words mean the distance from surface of the leiomyosarcoma. In **(b)**, words mean the points of myoma that ROIs were placed (figure of myoma was not shown).

**Figure 3:**
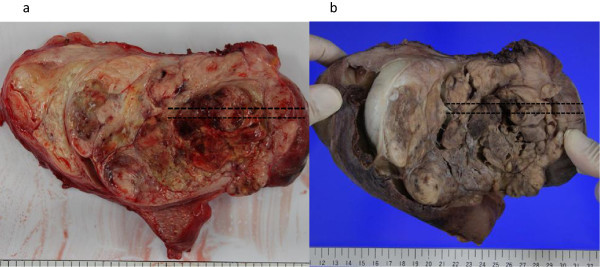
**Macroscopic findings of uterine leiomyosarcoma.** Section of resected tumor **a** and formalin-fixed sample **b**. Widespread necrosis is seen macroscopically, and its inner structure is heterogeneous as seen on gray-scale. The dotted line shows the region which matches the leiomyosarcoma indicated by the color scale as shown in Figure 1. An incision was made along the dotted line and comparison was made with pathological findings and VTQ study.

**Figure 4:**
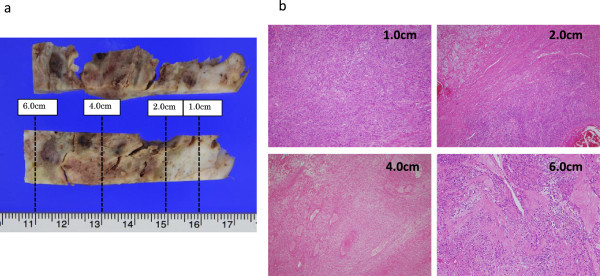
**Comparison of VTQ and pathological findings in case of leiomyosarcoma. a**: Formalin-fixed samples of the sections of 1.0 cm, 2.0 cm, 4.0 cm and 6.0 cm from each tumor surface where ROIs were placed. **b**: Histopathological findings of the sections were studied.

Virtual tissue quantification (VTQ) study and virtual tissue imaging (VTI) were performed preoperatively by one gynecologist. An ACUSON S2000 ultrasound (Mochida Siemens Medical Systems Co., Ltd, Tokyo, Japan) was used. The tip of the probe was positioned 2 cm below the navel, perpendicular to the floor. As for VTQ, regions of interest (ROIs) of 6 × 10 mm were placed on the median line of the tumor at 1 cm, 2 cm, 4 cm and 6 cm from the tumor surface, and the shear wave velocity (Vs, m/s) was measured five times. The mean ± SD of the Vs was calculated. The Vs was statistically analyzed using one-way ANOVA with Tukey’s post hoc test, and P < 0.05 was considered significant. Furthermore, the sections of the tumor where ultrasound was irradiated were examined macroscopically and histopathologically. The Vs and pathological findings in the four ROIs were compared. The ratios of viable cells, necrosis, fibrosis or hyaline degeneration in the region were evaluated for pathological findings. Statistical analysis was performed using SPSS software for Windows (SPSS Japan Inc., Tokyo).In VTI, irregular distribution was observed, suggesting a heterogeneous inner structure (Figure 1a). In the VTQ study, a multiple comparison test demonstrated that the Vs measured in the four ROIs were significantly different (4.14 ± 0.22, 3.71 ± 0.86, 1.55 ± 0.26, 2.19 ± 0.04), which also suggested a heterogeneous inner structure (Figure 2). Macroscopic findings of the sections where the aforesaid studies were performed are shown in Figure 3. The first 2 cm from the tumor surface was white, elastic, soft, and regarded as highly viable. The layer of 2 to 5 cm from the surface was yellowish-white, fragile, and had necrosis. In the tissue deeper than 5 cm, viable and necrotic components were mixed. Macroscopic and histological findings at the four measurement points are shown in Figure 4. The layer of 1 or 2 cm from the tumor surface showed severe dyskaryosis and mitosis. At 4 cm, necrosis was mainly observed.

At 6 cm, viable tissues, fibrosis and necrosis were mixed. Comparison between VTQ and pathological findings are shown in Table 1. The highest Vs value was obtained at 1 cm from the surface, where viable tissues accounted for 90% of all tissues. The lowest Vs value was measured at 4 cm from the surface, where coagulation necrosis accounted for 90%, showing a correlation with the histopathological findings.

**Table 1:** **Vs values at the four points, and the ratios of viability, necrosis, and fibrosis in the tissues**

	1.0 cm	2.0 cm	4.0 cm	6.0 cm
Vs(m/s)	4.14 ± 0.22	3.71 ± 0.66	1.55 ± 0.26	2.19 ± 0.04
Viability	90%	50%	0%	20%
Necrosis	0%	5%	90%	10%
Fibrosis	10%	45%	10%	70%

The highest Vs value was measured in the section of 1.0 cm depth where viable tissues accounted for 90%. The lowest was in the section of 4.0 cm depth where necrosis accounted for 90%.

### Case 2

A fifty-year-old woman visited a local doctor complaining of menorrhagia and was diagnosed with uterine leiomyoma. She was referred to our hospital for medical treatment. Gray-scale imaging revealed multiple low echoic myoma nuclei and clear margins. On MRI, the uterus, approximately 15 cm in size, had multiple low intensity masses with clear margins, and leiomyoma was diagnosed preoperatively. No abnormality was detected in vaginal and endometrial cytology, and tumor markers were within normal ranges. Hysterectomy was performed. Postoperative histopathological examination showed no mitosis or necrosis in the proliferating smooth muscle cells, and a diagnosis of leiomyoma was made. VTI and VTQ were preoperatively performed. As the number of leiomyomas was high and they were homogeneous, four ROIs were placed randomly in leiomyomas for VTQ. Measurement, calculation and comparison of the measurement results were performed as with Case1.The findings on VTI in Case2 are shown in Figure 1b. Gray-scale imaging revealed multiple myomas,and red on the color scale indicated their stiffness. Homogeneous inner structure was suggested as no significant differences were observed in the four ROIs (4.07 ± 0.36, 3.77 ± 0.66, 4.10 ± 0.40, 3.88 ± 0.73) in the VTQ study (Figure 2).

## Discussion

Uterine leiomyosarcoma is a rare malignant tumor which occurs in 0.64/100,000 women (Harlow et al. 1986), presents as a mass with unclear margins, causing macroscopic hemorrhage and necrosis (Wilkinson & Rollason 2001). It easily metastasizes hematogenously (Wilkinson & Rollason 2001) and is highly malignant. Differentiating leiomyosarcoma from leiomyoma on diagnostic imaging such as MRI is difficult (Murase et al. 1999), and its diagnosis is often made by histopathology of operated specimen. Hata et al. examined the color Doppler findings of five uterine sarcoma and 41 leiomyoma cases and reported that the usefulness of color Doppler for the differentiation of uterine sarcoma as the peak systolic velocity in intratumoral vessels is significantly high in uterine sarcoma (Hata et al. 1997). Exacoustous et al. retrospectively studied the ultrasound findings of eight uterine leiomyosarcoma and 21 leiomyoma cases, and reported that uterus volume, the diameter of the mass, and the presence or absence of cystic degeneration etc. are useful for differentiation using gray scale (Exacoustous et al. 2007). However, a definitive ultrasonographic diagnosis has not been established.

Recently, elastography has been developed as a non-invasive diagnostic tool. In superficial tumors, some studies demonstrated the usefulness of elastography for the differentiation of benignity and malignancy. Aly et al. and Gheonea et al. retrospectively studied the elastographic findings of 100 (Aly et al. 2010) and 85 (Gheonea et al. 2011) mammary tumor cases, respectively, and reported the usefulness of elastography for the differentiation of benignity and malignancy in mammary tumors. In thyroid examination, many studies have demonstrated that elastography has a sensitivity and specificity of 90% and is useful for detecting malignant tumors (Sebag et al. 2010; Rubaltelli et al. 2009; Asteria et al. 2008). However, there have been no reports on the use of elastography for the diagnosis of gynecologic tumors.

In our study, we performed VTI and VTQ for two patients with uterine smooth muscle tumors. VTI and VTQ showed a heterogeneous structure with viable tissue and necrotic tissue in the leiomyosarcoma, and the leiomyoma was shown as hard on the whole. The Vs at four points in the leiomyosarcoma was significantly different, whereas no significant differences were observed in the leiomyoma. The highest Vs was measured in the highly viable region of the leiomyosarcoma, and the lowest was in the severely necrotic region. Furthermore, the results obtained from VTQ agreed with macroscopic findings and palpable hardness. From the above, our VTQ findings were consistent with macroscopic and histopathological findings in the leiomyosarcoma.

VTQ is a technique to measure tissue stiffness using elastic shear waves (Nightingale et al. 2003). There are many reports stating that VTQ demonstrates stiffness of malignant tumors or inflammation (Goertz et al. 2010; Mateen et al. 2012; Bojunga et al. 2012; Bai et al. 2012). Our VTQ study revealed that leiomyosarcoma is soft and heterogeneous while leiomyoma is hard, being consistent with macroscopic and histopathological findings. Especially, the Vs was low in coagulation necrosis, which is the most pathognomonic feature of uterine leiomyosarcoma (Wilkinson & Rollason 2001). There have been no reports of elastography performed for differential diagnosis of gynecologic tumors such as uterine smooth muscle tumors. We performed VTQ in one leiomyosarcoma case and one leiomyoma case and obtained significant differences. In addition, we found correlations between Vs and histopathological findings in leiomyosarcoma. Although the differentiation of leiomyoma and leiomyosarcoma may be difficult only by VTI and VTQ, these findings have possibility to suggest that VTQ can be useful for the differentiation of uterine smooth muscle tumor.

### Human rights statements and informed consent

All procedures followed were in accordance with the ethical standards of the responsible committee on human experimentation (institutional and national) and with the Helsinki Declaration of 1975, as revised in 2008. Informed consent was obtained from all patients for being included in the study.
